# Supplementary material for: Zeb2 regulates differentiation of long-lived effector of invariant natural killer T cells
Source: Commun Biol. 2023 Oct 30;6:1070. doi: 10.1038/s42003-023-05421-w (PMC10616117; doi:10.1038/s42003-023-05421-w)
Supplement: Supplementary file 2 — Supplementary Figures and Table [file 42003_2023_5421_MOESM2_ESM.pdf]

Supplementary Figure 1

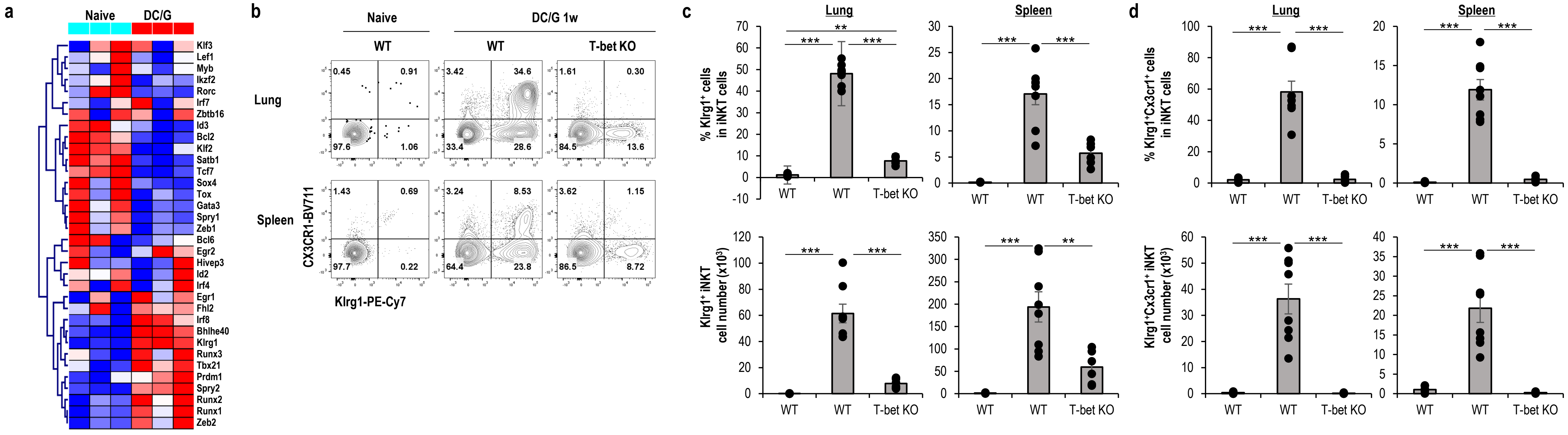

Supplementary Fig. 1 T-bet regulates Zeb2 expression, followed by induction of Klrg1^{+} iNKT cells.

a. As in **Fig. 1a**, but the heatmap of expression of the indicated TFs in lung iNKT cells. **b.** WT and T-bet KO mice were immunized with DC/Gal 1 week before. Representative dot plots show the Cx3cr1 and Klrg1 expression of lung iNKT cells. **c.** The frequency (upper) and cell number (lower) of Klrg1^{+} cells in iNKT cells were summarized respectively. (n= 6-8, mean ± SEM, one experiment) **d.** The frequency (upper) and cell number (lower) of Klrg1^{+}Cx3cr1^{+} cells in iNKT cells were summarized respectively. (n= 6-8, mean ± SEM, one experiment) *p < 0.05, **p < 0.01, ***p < 0.001 ANOVA Tukey–Kramer method.

Supplementary Figure 2

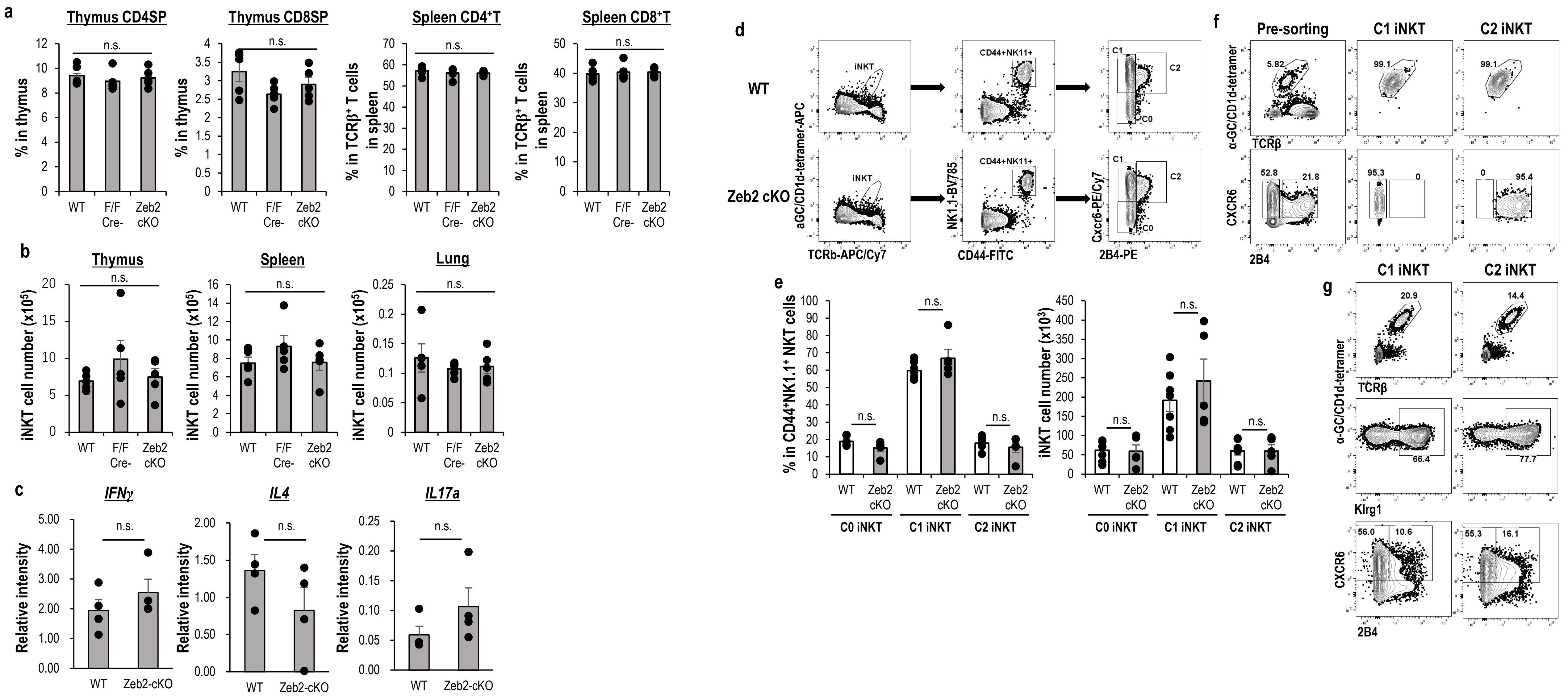

Supplementary Fig. 2 T cells and iNKT cells in WT, littermate, and Zeb2cKO mice.

a. The frequency of CD4+ T cells and CD8+ T cells in the thymus and spleen. . (n= 5, mean ± SEM, four independent experiments) n.s. Student's t-test **b.** The absolute cell number of iNKT cells in the thymus, spleen and lung. (n= 5, mean ± SEM, four independent experiments) n.s. Student's t-test **c**. Expression of *IFNγ*, *IL4* and *IL17a* in lung iNKT cells from naïve WT and Zeb2-cKO mice. Gene expression was analyzed by qPCR (n=4, mean ± SEM) n.s. Student's t-test **d, e.** The frequency of iNKT cells in the thymus. Three subsets, C0 (CXCR6-2B4-), C1 (CXCR6+2B4-) and C2 (CXCR6+2B4+) in CD44+NK1.1+ iNKT cells in the thymus from WT and Zeb2-cKO mice were analyzed. Representative dot plot of the gating pattern of these subsets **(c)** and the frequency of each subset **(d)** are summarized. (n= 5-7, mean ± SEM, four experiments). n.s. Student's t-test **f.** Sorting strategy of C1 and C2 iNKT cells of the thymus from WT mice. **g.** Induction of Klrg1+ iNKT cells from thymic C1 and C2 iNKT cell subset. C1 and C2 iNKT cells were sorted from WT mice. $3x10^5$ cells of each iNKT subset were transferred to Rag 1KO mice, respectively, and then immunized with DC/Gal 5 days later. Lung iNKT cells were analyzed 1 week after DC/Gal immunization (upper). Representative dot plot of the expression of Klrg1 (middle), 2B4 and CXCR6 (lower) in lung iNKT cells.

Supplementary Figure 3

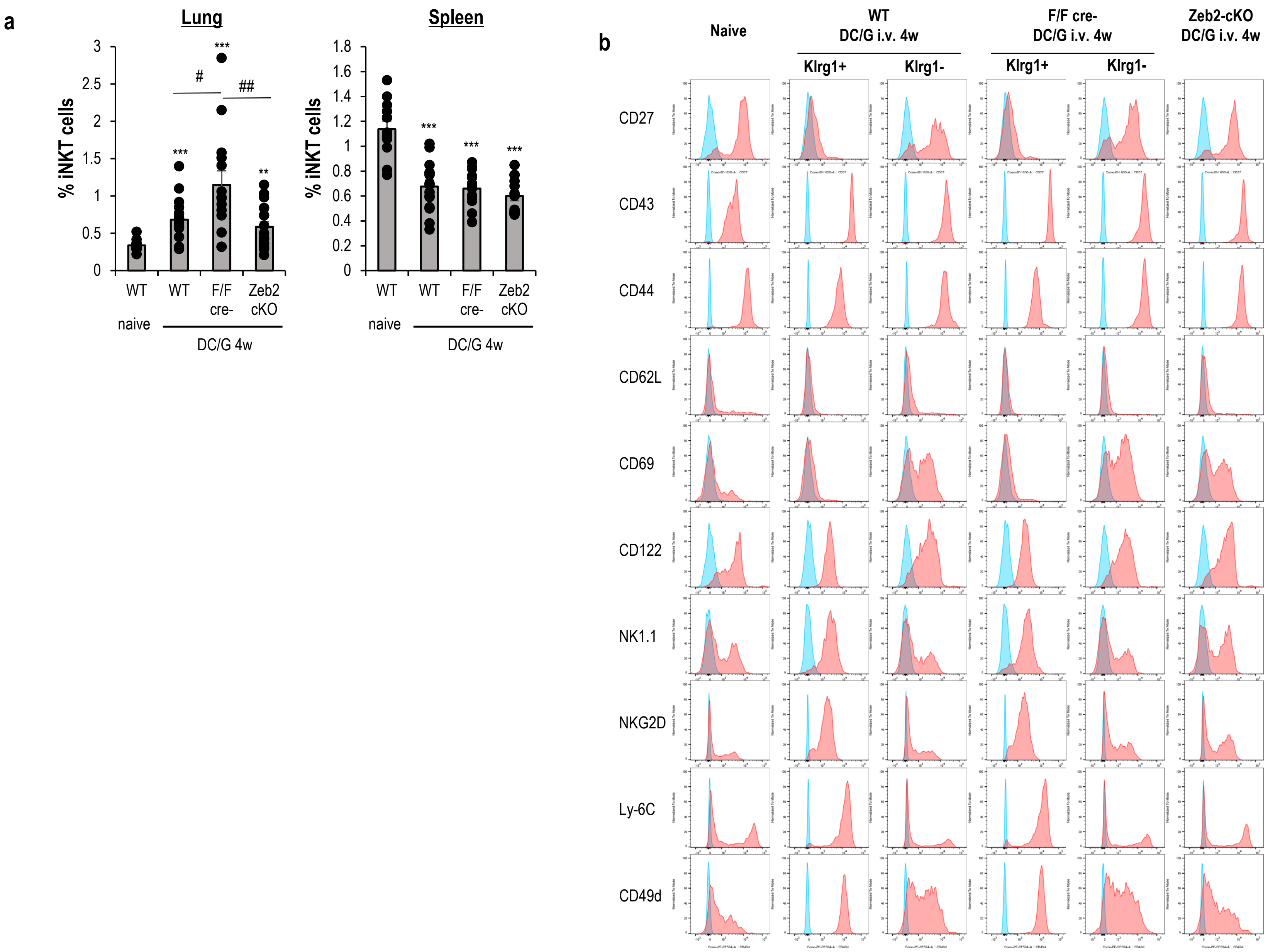

Supplementary Fig. 3 The frequency and phenotypes of iNKT cells in WT and Zeb2 cKO mice after DC/Gal immunization.
The WT, littermate, and Zeb2-cKO mice were immunized with DC/Gal. Four weeks later, lung and spleen iNKT cells were analyzed using flow cytometry.
a. The frequency of iNKT cells in the indicated groups (lung: n = 14-19, spleen: n=12-15, mean ± SEM). $**p < 0.01$, $***p < 0.001$ Student's *t*-test to WT naïve, $\#p < 0.05$, $\#\#p < 0.01$ ANOVA Tukey–Kramer method in the immunized group.
b. Expression of the indicated markers in whole lung iNKT cells of WT naïve mice, DC/Gal-immunized Zeb2-cKO mice or Klrg1^{+} iNKT and Klrg1^{-} iNKT cells of DC/Gal immunized WT or littermate mice. (isotype; blue, the indicated marker; red)

Supplementary Figure 4

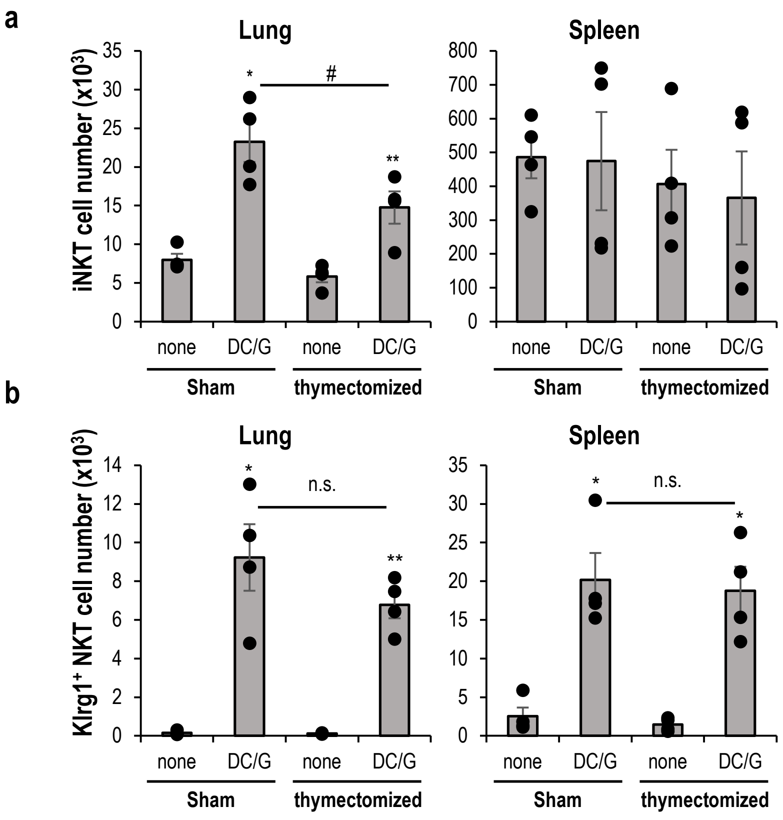

Supplementary Fig. 4 iNKT cells in the thymectomized mice.
Two weeks after thymectomy or sham treatment, the mice were immunized with DC/Gal. Four weeks later, lung and spleen iNKT cells were analyzed by flow cytometry. **a.** The cell number of iNKT cells in the indicated groups (n = 4, mean ± SEM). **b.** The cell number of Klrg1^{+} iNKT cells in the indicated groups (n = 4, mean ± SEM). **p* < 0.05, ***p* < 0.01 Student's *t*-test to none, #*p* < 0.05 Student's t-test to sham mice.

Supplementary Figure 5

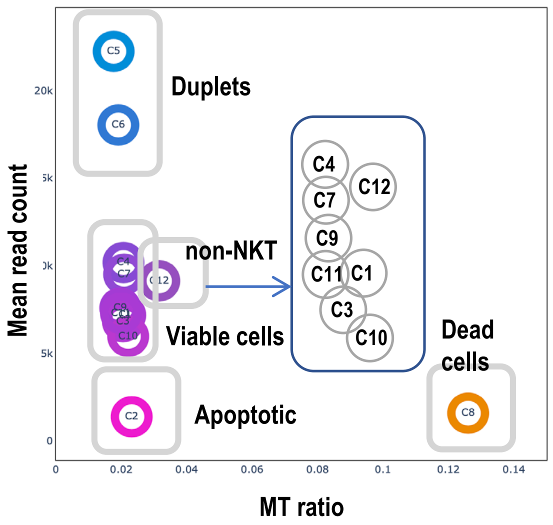

Supplementary Fig. 5 Single-cell transcriptome analysis of Klrg1^{+} iNKT cells

Lung Klrg1^{+} iNKT cells were sorted from DC/Gal-immunized WT and Zeb2-cKO mice and analyzed using sc-RNAseq. Cell state identification of each cluster was performed. Mean read counts and ratio of MT genes were calculated. Cell clusters with low gene counts were considered dead or apoptotic. Clusters with double expression read counts were assumed to have doublet cells. Clusters 5 and 6 appeared to be a mixture of two or more cells. Cluster 8 appeared to be dead cells based on the low read counts and Cluster 12 was a non-NKT population based on gene expression. Therefore, we excluded Clusters, 5, 6, 8 and 12 from further analysis.

Supplementary Figure 6

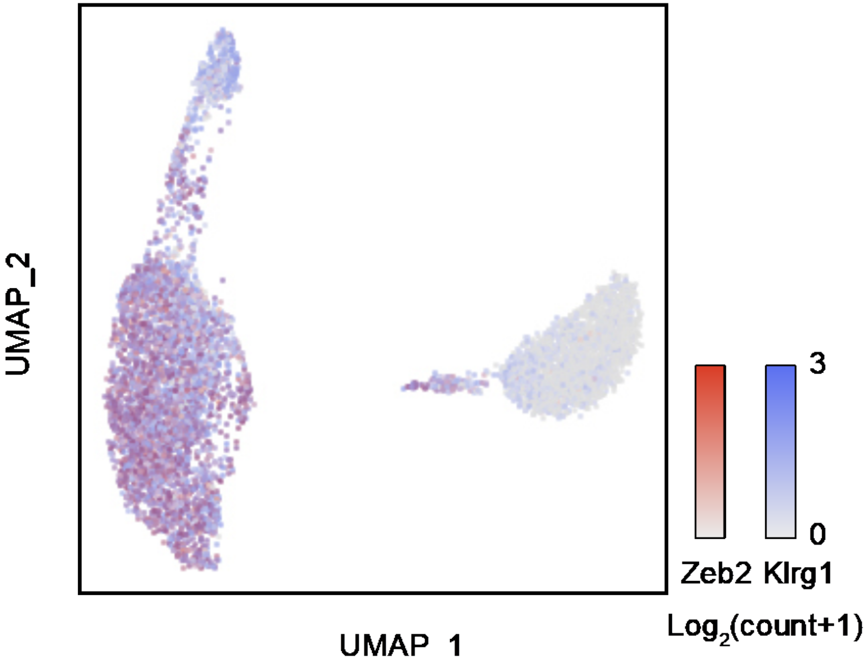

Supplementary Fig. 6 Klrg1 and Zeb2 expression of Klrg1$^+$ iNKT cells in sc-RNAseq analysis.

Similar to Supplementary Fig. 5, lung Klrg1$^+$ iNKT cells were sorted from DC/Gal-immunized WT and Zeb2-cKO mice and analyzed using sc-RNAseq. The expression of *Zeb2* and *Klrg1* is shown in the UMAP diagram and is indicated in red or blue according to the expression level.

Antibodies list

Clone and specificity	Supplier	Catalog number	Lot number	Antibody dilution
EPR3915 (PE) [rabbit anti-mouse Glut1]	Abcam	Cat# ab209449	Lot# GR3237858-3	1:500
GK1.5 (AlexaFluoro700) [rat anti-mouse CD4]	Biolegend	Cat# 100430	Lot# B313203	1:100
GK1.5 (BUV395) [rat anti-mouse CD4]	BD Biosciences	Cat# 563790	Lot# 1256599	1:200
53-6.7 (APC-Cy7) [rat anti-mouse CD8]	Biolegend	Cat# 100714	Lot# B318900	1:1000
53-6.7 (BUV395) [rat anti-mouse CD8]	BD Biosciences	Cat# 563786	Lot# 1097464	1:200
53-6.7 (BUV737) [rat anti-mouse CD8]	BD Biosciences	Cat# 564297	Lot# 7165972	1:400
M17/4 (FITC) [rar anti-mouse/human CD11a]	TermoFisher Scientific	Cat# 11-0111-82	Lot# E00144-1630	1:500
M1/70 (APC-Fire) [rat anti-mouse/human CD11b]	Biolegend	Cat# 101262	Lot# B262767	1:1000
M1/70 (BUV737) [rat anti-mouse/human CD11b]	BD Biosciences	Cat# 564443	Lot# 7061691	1:400
93 (purified) [rat anti-mouse CD16/32]	Biolegend	Cat# 101302	Lot# B366439	1:100
1D3 (PerCP-Cy5.5) [rat anti-mouse CD19]	TermoFisher Scientific	Cat# 45-0193-82	Lot# 4300341	1:200
6D5 (Pacific Blue) [rat anti-mouse CD19]	Biolegend	Cat# 115523	Lot# B265434	1:200
M1/69 (PerCP-Cy5.5) [rat anti-mouse CD24]	Bioligend	Cat# 101824	Lot# B266796	1:500
LG.3A10 (biotin) [hamster anti-mouse CD27]	BD Biosciences	Cat# 558753	Lot# 6015550	1:100
S11 (PE/Dazzle) [rat anti-mouse CD43]	BioLegend	Cat# 143218	Lot# B297552	1:200
1M7 (FITC) [rat anti-mouse CD44]	BD Biosciences	Cat# 553133	Lot# 6111755	1:1000
30-F11 (FITC) [rat anit-mouse CD45]	BioLegend	Cat# 103108	Lot# B266197	1:500
30-F11 (BV510) [rat anti-mouse CD45]	BD Biosciences	Cat# 563891	Lot#1169522	1:500
R1-2 (PE/Dazzle) [rat anti-mouse CD49d]	BioLegend	Cat# 103626	Lot# B291587	1:200
MEL-14 (BUV737) [rat anti-mouse CD62L]	BD Biosciences	Cat# 612833	Lot# 9263599	1:200
H1.2F3 (BV711) [hamster anti-mouse CD69]	BioLegend	Cat# 104537	Lot# B266676	1:200
H1.2F3 (PE-Cy7) [hamster anti-mouse CD69]	BioLegend	Cat# 104512	Lot# 282385	1:200
2E7 (PE-Cy7) [hamster anti-mouse CD103]	Biolegend	Cat# 121425	Lot# B214516	1:500
TM-BETA1 (FITC) [rat anti-mouse CD122]	BD Biosciences	Cat# 553361	Lot# 4283922	1:500
A7R34 (PE) [rat anti-mouse CD127]	TermoFisher Scientific	Cat# 12-1271-82	Lot# E01470-1630	1:200
SB/199 (BUV737) [rat anti-mouse CD127]	BD Biosciences	Cat# 564399	Lot# 8303595	1:200
eBIO244F4 (PE) [rat anti-mouse 2B4]	TermoFisher Scientific	Cat# 12-2441-82	Lot# E01612-1630	1:200
m2B4 (PE) [mouse anti-mouse 2B4]	Biolegend	Cat# 133508	Lot# B294046	1:200
29F.1A12 (BV421) [rat anti-mouse CD279]	Biolegend	Cat# 135218	Lot# B349792	1:50
29-2L17 (PE) [hamster anti-mouse CCR6]	BioLegend	Cat# 129804	Lot# B257642	1:200
L138D7 (biotin) [rat anti-mouse CXCR5]	Biolegend	Cat# 145510	Lot# B348507	1:500
SA051D1 (PE-Cy7) [rat anti-mouse CXCR6]	Biolegend	Cat# 151119	Lot# B377024	1:200
SA011F11 (BV711) [mouse anti-mouse CX3CR1]	Biolegend	Cat# 149031	Lot# B364019	1:200
PK136 (BV785) [mouse anti-mouse NK1.1]	Biolegend	Cat# 108749	Lot# B279624	1:500
CX5 (PE) [rat anti-mouse NKG2D]	TermoFisher Scientific	Cat# 12-5882-82	Lot# E01870-1637	1:200
HK1.4 (FITC) [rat anti-mouse Ly-6C]	BioLegend	Cat# 128006	Lot# B247728	1:1000
2F1 (PerCP-Cy5.5) [hamster anti-mouse/human KLRG1]	Biolegend	Cat# 138418	Lot# B269979	1:400
2F1 (PE-Cy7) [hamster anti-mouse KLRG1]	TermoFisher Scientific	Cat# 25-5893-82	Lot# 4300747	1:400
2F1 (BV785) [hamster anti-mouse KLRG1]	Biolegend	Cat# 138429	Lot# B340352	1:400
2F1 (BUV395) [hamster anti-mouse KLRG1]	BD Biosciences	Cat# 740279	Lot# 0083180	1:200
H57-597 (Pacific Blue) [hamster anti-mouse TCRbeta]	Biolegend	Cat# 109226	Lot# B348544	1:200
H57-597 (APC-Cy7) [hamster anti-mouse TCRbeta]	Biolegend	Cat# 109220	Lot# B361176	1:200
SolA15 (FITC) [rat anti-mouse Ki-67]	TermoFisher Scientific	Cat# 11-5698-82	Lot# 2191034	1:400
3G8.5 (PE) [mouse anti-mouse granzyme A]	Santa cruz	Cat# sc-33692 PE	Lot# B0112	1:1
eBio4B10 (PE-Cy7) [mouse anti-mouse T-bet]	TermoFisher Scientific	Cat# 25-5825-82	Lot# 4277988	1:100
TWAJ (PE-Cy7) [rat anti-mouse Gata3]	TermoFisher Scientific	Cat# 25-9966-42	Lot# 4304262	1:100
Q31-378 (BV650) [mouse anti-mouse RORgamma-t]	BD Biosciences	Cat# 564722	Lot# 7096784	1:100
9E12(PE) [hamster anti-mouse PLZF]	BioLegend	Cat# 145804	Lot# B181991	1:200
S33-966 (PE) [mouse anti-mouse TCF-7/TCF-1]	BD Biosciences	Cat# 564217	Lot# 8144533	1:100
5E7 (PE-CF594) [rat anti-mouse Blimp-1]	BD Biosciences	Cat# 564269	Lot# 2011596	1:100
