# Supplementary material for: Zeb2 regulates differentiation of long-lived effector of invariant natural killer T cells
Source: Commun Biol. 2023 Oct 30;6:1070. doi: 10.1038/s42003-023-05421-w (PMC10616117; doi:10.1038/s42003-023-05421-w)
Supplement: Supplementary file 5 — Reporting summary [file 42003_2023_5421_MOESM5_ESM.pdf]

nature portfolio

Corresponding author(s): Shin-ichiro Fujii

Last updated by author(s): 2023/09/29

Reporting Summary

Nature Portfolio wishes to improve the reproducibility of the work that we publish. This form provides structure for consistency and transparency in reporting. For further information on Nature Portfolio policies, see our Editorial Policies and the Editorial Policy Checklist.

Statistics

For all statistical analyses, confirm that the following items are present in the figure legend, table legend, main text, or Methods section.

n/a	Confirmed	
☐	☒	The exact sample size (*n*) for each experimental group/condition, given as a discrete number and unit of measurement
☐	☒	A statement on whether measurements were taken from distinct samples or whether the same sample was measured repeatedly
☐	☒	The statistical test(s) used AND whether they are one- or two-sided<br>*Only common tests should be described solely by name; describe more complex techniques in the Methods section.*
☒	☐	A description of all covariates tested
☐	☒	A description of any assumptions or corrections, such as tests of normality and adjustment for multiple comparisons
☐	☒	A full description of the statistical parameters including central tendency (e.g. means) or other basic estimates (e.g. regression coefficient) AND variation (e.g. standard deviation) or associated estimates of uncertainty (e.g. confidence intervals)
☐	☒	For null hypothesis testing, the test statistic (e.g. *F*, *t*, *r*) with confidence intervals, effect sizes, degrees of freedom and *P* value noted<br>*Give P values as exact values whenever suitable.*
☒	☐	For Bayesian analysis, information on the choice of priors and Markov chain Monte Carlo settings
☒	☐	For hierarchical and complex designs, identification of the appropriate level for tests and full reporting of outcomes
☒	☐	Estimates of effect sizes (e.g. Cohen's *d*, Pearson's *r*), indicating how they were calculated

Our web collection on statistics for biologists contains articles on many of the points above.

Software and code

Policy information about availability of computer code

Data collection	BD FACSDivaTM Software v.8.0.1 (Becton Dickinson & Company)
Data analysis	Flow jo v10.8 (Becton Dickinson & Company)<br>StatMate V (Nihon 3B Scientific Inc.)<br>Trimgalore software (version 0.6.7), Kallisto (version 0.48.0) and DESeq2 algorithm (version 1.38.2) for RNA-seq<br>10 x Genomics cellranger (version 6.1.2) and Seurat toolkit(version 4.0.3) for single cell transcriptome analysis.<br>Trajectories among NKT1 clusters were analyzed using pyslingshot (version 0.0.2, https://github.com/mossjacob/pyslingshot), a Python implementation of Slingshot (version 2.7.0, https://github.com/kstreet13/slingshot).<br>Other code for data cleaning and analysis is provided as part of the replication package. It is available at https://github.com/takaho/nktsc for review.

For manuscripts utilizing custom algorithms or software that are central to the research but not yet described in published literature, software must be made available to editors and reviewers. We strongly encourage code deposition in a community repository (e.g. GitHub). See the Nature Portfolio guidelines for submitting code & software for further information.

Data

Policy information about availability of data

All manuscripts must include a data availability statement. This statement should provide the following information, where applicable:

- Accession codes, unique identifiers, or web links for publicly available datasets
- A description of any restrictions on data availability
- For clinical datasets or third party data, please ensure that the statement adheres to our policy

RNA-seq data that support the findings of this study have been deposited in the Gene Expression Omnibus (GEO) database with accession codes GSE12806917. The scRNA-seq data of iNKT cells have been deposited with links to BioProject accession number PRJNA1021694 in the DDBJ BioProject database. All source data underlying the graphs presented in the main figures are available in Supplementary Data 1.

Human research participants

Policy information about studies involving human research participants and Sex and Gender in Research.

Reporting on sex and gender	*Use the terms sex (biological attribute) and gender (shaped by social and cultural circumstances) carefully in order to avoid confusing both terms. Indicate if findings apply to only one sex or gender; describe whether sex and gender were considered in study design whether sex and/or gender was determined based on self-reporting or assigned and methods used. Provide in the source data disaggregated sex and gender data where this information has been collected, and consent has been obtained for sharing of individual-level data; provide overall numbers in this Reporting Summary. Please state if this information has not been collected. Report sex- and gender-based analyses where performed, justify reasons for lack of sex- and gender-based analysis.*
Population characteristics	*Describe the covariate-relevant population characteristics of the human research participants (e.g. age, genotypic information, past and current diagnosis and treatment categories). If you filled out the behavioural & social sciences study design questions and have nothing to add here, write "See above."*
Recruitment	*Describe how participants were recruited. Outline any potential self-selection bias or other biases that may be present and how these are likely to impact results.*
Ethics oversight	*Identify the organization(s) that approved the study protocol.*

Note that full information on the approval of the study protocol must also be provided in the manuscript.

Field-specific reporting

Please select the one below that is the best fit for your research. If you are not sure, read the appropriate sections before making your selection.

☒ Life sciences ☐ Behavioural & social sciences ☐ Ecological, evolutionary & environmental sciences

For a reference copy of the document with all sections, see nature.com/documents/nr-reporting-summary-flat.pdf

Life sciences study design

All studies must disclose on these points even when the disclosure is negative.

Sample size	Sample size was determined by referring to recently published related papers.
Data exclusions	No data was excluded from the analyses except the limitation of cell numbers.
Replication	Basically each experiment was repeated more than two times.
Randomization	No randomization was performed.
Blinding	No blinding was performed.

Reporting for specific materials, systems and methods

We require information from authors about some types of materials, experimental systems and methods used in many studies. Here, indicate whether each material, system or method listed is relevant to your study. If you are not sure if a list item applies to your research, read the appropriate section before selecting a response.

Materials & experimental systems

n/a	Involved in the study
☐	☒ Antibodies
☒	☐ Eukaryotic cell lines
☒	☐ Palaeontology and archaeology
☐	☒ Animals and other organisms
☒	☐ Clinical data
☒	☐ Dual use research of concern

Methods

n/a	Involved in the study
☒	☐ ChIP-seq
☐	☒ Flow cytometry
☒	☐ MRI-based neuroimaging

Antibodies

Antibodies used

EPR3915 (PE) [rabbit anti-mouse Glut1] Abcam Cat# ab209449 Lot# GR3237858-3
GK1.5 (AlexaFluoro700) [rat anti-mouse CD4] Biolegend Cat# 100430 Lot# B313203
GK1.5 (BUV395) [rat anti-mouse CD4] BD Biosciences Cat# 563790 Lot# 1256599
53-6.7 (APC-Cy7) [rat anti-mouse CD8] Biolegend Cat# 100714 Lot# B318900
53-6.7 (BUV395) [rat anti-mouse CD8] BD Biosciences Cat# 563786 Lot# 1097464
53-6.7 (BUV737) [rat anti-mouse CD8] BD Biosciences Cat# 564297 Lot# 7165972
M17/4 (FITC) [rar anti-mouse/human CD11a] TermoFisher Scientific Cat# 11-0111-82 Lot# E00144-1630
M1/70 (APC-Fire) [rat anti-mouse/human CD11b] Biolegend Cat# 101262 Lot# B262767
M1/70 (BUV737) [rat anti-mouse/human CD11b] BD Biosciences Cat# 564443 Lot# 7061691
93 (purified) [rat anti-mouse CD16/32] Biolegend Cat# 101302 Lot# B366439
1D3 (PerCP-Cy5.5) [rat anti-mouse CD19] TermoFisher Scientific Cat# 45-0193-82 Lot# 4300341
6D5 (Pacific Blue) [rat anti-mouse CD19] Biolegend Cat# 115523 Lot# B265434
M1/69 (PerCP-Cy5.5) [rat anti-mouse CD24] Bioligend Cat# 101824 Lot# B266796
LG.3A10 (biotin) [hamster anti-mouse CD27] BD Biosciences Cat# 558753 Lot# 6015550
S11 (PE/Dazzle) [rat anti-mouse CD43] BioLegend Cat# 143218 Lot# B297552
1M7 (FITC) [rat anti-mouse CD44] BD Biosciences Cat# 553133 Lot# 6111755
30-F11 (FITC) [rat anit-mouse CD45] BioLegend Cat# 103108 Lot# B266197
30-F11 (BV510) [rat anti-mouse CD45] BD Biosciences Cat# 563891 Lot#1169522
R1-2 (PE/Dazzle) [rat anti-mouse CD49d] BioLegend Cat# 103626 Lot# B291587
MEL-14 (BUV737) [rat anti-mouse CD62L] BD Biosciences Cat# 612833 Lot# 9263599
H1.2F3 (BV711) [hamster anti-mouse CD69] BioLegend Cat# 104537 Lot# B266676
H1.2F3 (PE-Cy7) [hamster anti-mouse CD69] BioLegend Cat# 104512 Lot# 282385
2E7 (PE-Cy7) [hamster anti-mouse CD103] Biolegend Cat# 121425 Lot# B214516
TM-BETA1 (FITC) [rat anti-mouse CD122] BD Biosciences Cat# 553361 Lot# 4283922
A7R34 (PE) [rat anti-mouse CD127] TermoFisher Scientific Cat# 12-1271-82 Lot# E01470-1630
SB/199 (BUV737) [rat anti-mouse CD127] BD Biosciences Cat# 564399 Lot# 8303595
eBIO244F4 (PE) [rat anti-mouse 2B4] TermoFisher Scientific Cat# 12-2441-82 Lot# E01612-1630
m2B4 (PE) [mouse anti-mouse 2B4] Biolegend Cat# 133508 Lot# B294046
29F.1A12 (BV421) [rat anti-mouse CD279] Biolegend Cat# 135218 Lot# B349792
29-2L17 (PE) [hamster anti-mouse CCR6] BioLegend Cat# 129804 Lot# B257642
L138D7 (biotin) [rat anti-mouse CXCR5] Biolegend Cat# 145510 Lot# B348507
SA051D1 (PE-Cy7) [rat anti-mouse CXCR6] Biolegend Cat# 151119 Lot# B377024
SA011F11 (BV711) [mouse anti-mouse CX3CR1] Biolegend Cat# 149031 Lot# B364019
PK136 (BV785) [mouse anti-mouse NK1.1] Biolegend Cat# 108749 Lot# B279624
CX5 (PE) [rat anti-mouse NKG2D] TermoFisher Scientific Cat# 12-5882-82 Lot# E01870-1637
HK1.4 (FITC) [rat anti-mouse Ly-6C] BioLegend Cat# 128006 Lot# B247728
2F1 (PerCP-Cy5.5) [hamster anti-mouse/human KLRG1] Biolegend Cat# 138418 Lot# B269979
2F1 (PE-Cy7) [hamster anti-mouse KLRG1] TermoFisher Scientific Cat# 25-5893-82 Lot# 4300747
2F1 (BV785) [hamster anti-mouse KLRG1] Biolegend Cat# 138429 Lot# B340352
2F1 (BUV395) [hamster anti-mouse KLRG1] BD Biosciences Cat# 740279 Lot# 0083180
H57-597 (Pacific Blue) [hamster anti-mouse TCRbeta] Biolegend Cat# 109226 Lot# B348544
H57-597 (APC-Cy7) [hamster anti-mouse TCRbeta] Biolegend Cat# 109220 Lot# B361176
SolA15 (FITC) [rat anti-mouse Ki-67] TermoFisher Scientific Cat# 11-5698-82 Lot# 2191034
3G8.5 (PE) [mouse anti-mouse granzyme A] Santa cruz Cat# sc-33692 PE Lot# B0112
eBio4B10 (PE-Cy7) [mouse anti-mouse T-bet] TermoFisher Scientific Cat# 25-5825-82 Lot# 4277988
TWAJ (PE-Cy7) [rat anti-mouse Gata3] TermoFisher Scientific Cat# 25-9966-42 Lot# 4304262
Q31-378 (BV650) [mouse anti-mouse RORgamma-t] BD Biosciences Cat# 564722 Lot# 7096784
9E12(PE) [hamster anti-mouse PLZF] BioLegend Cat# 145804 Lot# B181991
S33-966 (PE) [mouse anti-mouse TCF-7/TCF-1] BD Biosciences Cat# 564217 Lot# 8144533
5E7 (PE-CF594) [rat anti-mouse Blimp-1] BD Biosciences Cat# 564269 Lot# 2011596

Validation

All antibodies were purchased from the above stated companies. Antibodies are well described and published elsewhere. Informations can be sought from the manufacures website under catalogue number.

Animals and other research organisms

Policy information about studies involving animals; ARRIVE guidelines recommended for reporting animal research, and Sex and Gender in Research

Laboratory animals	In this study, we used C57BL/6 mice and transgenic mice with same background. All mice were used at 6 to 12 week after birth.
Wild animals	We did not use wild animals.
Reporting on sex	Though we mainly used female mice in our experiments as easier for handling, we confirmed similarity between male and female in critical evidence of our study.
Field-collected samples	We did not use field-collected samples.
Ethics oversight	All procedures were performed in compliance with the protocols approved by the Institutional Animal Care Committee at RIKEN.

Note that full information on the approval of the study protocol must also be provided in the manuscript.

Flow Cytometry

Plots

Confirm that:

☒ The axis labels state the marker and fluorochrome used (e.g. CD4-FITC).

☒ The axis scales are clearly visible. Include numbers along axes only for bottom left plot of group (a 'group' is an analysis of identical markers).

☒ All plots are contour plots with outliers or pseudocolor plots.

☒ A numerical value for number of cells or percentage (with statistics) is provided.

Methodology

Sample preparation	For preparation of single cell from lymphoid organs, thymus and spleen were homogenized through a 70 µm cell strainer and erythrocytes were lysed with ACK lysing buffer. In the case of non-lymphoid organs, lung was digested with collagenase D and mononuclear cells were separated by Percoll gradients (40/70%).
Instrument	BD LSR Fortessa X-20, BD FACS ARIA III Cell Sorter
Software	Data was acquired with the Diva software (BD Bioscience) and analyzed with FlowJo v10.8 (BD Bioscience)
Cell population abundance	Cells populations were reported as a proportion of a specific population (% Klrg1+ cells in iNKT etc).
Gating strategy	Lymphocytes were selected in the FSC/SSC gate. Single cell were selected. Viable cells were determined using Aqua live/dead (Invitrogen). iNKT cells were detected as alpha-galactosylceramide-loaded CD1d-tetramer+ T-cell receptor beta-chain+ cells. iNKT cells were further divided by indicated markers.

☐ Tick this box to confirm that a figure exemplifying the gating strategy is provided in the Supplementary Information.
